# The Role of Chemokines in Hepatitis C Virus-Mediated Liver Disease

**DOI:** 10.3390/ijms15034747

**Published:** 2014-03-18

**Authors:** Anette Brass, Erwin Daniel Brenndörfer

**Affiliations:** Division of Clinical Microbiology, Department of Laboratory Medicine, Karolinska Institutet, Stockholm 14186, Sweden; E-Mail: anette.brass@gmx.de

**Keywords:** HCV, intrahepatic immunity, immune escape, liver fibrosis, liver cancer, hepatitis C treatment

## Abstract

The hepatitis C virus (HCV) is a global health problem affecting more than 170 million people. A chronic HCV infection is associated with liver fibrosis, liver cirrhosis and hepatocellular carcinoma. To enable viral persistence, HCV has developed mechanisms to modulate both innate and adaptive immunity. The recruitment of antiviral immune cells in the liver is mainly dependent on the release of specific chemokines. Thus, the modulation of their expression could represent an efficient viral escape mechanism to hamper specific immune cell migration to the liver during the acute phase of the infection. HCV-mediated changes in hepatic immune cell chemotaxis during the chronic phase of the infection are significantly affecting antiviral immunity and tissue damage and thus influence survival of both the host and the virus. This review summarizes our current understanding of the HCV-mediated modulation of chemokine expression and of its impact on the development of liver disease. A profound knowledge of the strategies used by HCV to interfere with the host’s immune response and the pro-fibrotic and pro-carcinogenic activities of HCV is essential to be able to design effective immunotherapies against HCV and HCV-mediated liver diseases.

## 1. Introduction

The hepatitis C virus (HCV) is very successful at establishing a chronic infection that leads over decades to the development of severe liver diseases including fibrosis, cirrhosis and hepatocellular carcinoma. This review starts by describing shortly how HCV evades/modulates the innate immune response and by giving an overview of the current treatment for chronic hepatitis C. Then, some general information about chemokines is presented followed by the description of the role of chemokines in intrahepatic immunity. In the following chapters, we describe in detail our current understanding of how HCV modulates the chemokine response both with regard to altered recruitment of immune cells and impact on the development of HCV-mediated liver diseases such as fibrosis and HCC. Finally, we discuss the potential of therapies based on the amplification or interference of chemokine signaling for liver-related diseases.

### 1.1. Hepatitis C Virus

Infection with HCV is a global health problem affecting about 170 million people worldwide [1]. About 70%–80% of those infected with HCV develop a chronic infection [2,3], which is a major cause for severe liver disease including fibrosis, cirrhosis and hepatocellular carcinoma (HCC) [4].

HCV belongs to the genus *Hepacivirus* and is a member of the *Flaviviridae* family. The virus has a positive single strand RNA genome of 9.6 kb that encodes for a polyprotein, which is cleaved into three structural proteins (core, E1, E2) and seven non-structural (NS) proteins (p7, NS2, NS3, NS4A, NS4B, NS5A, NS5B) by host and viral proteases [5,6]. Due to the lack of a proofreading function of the viral RNA-dependent RNA polymerase NS5B, HCV has a high genetic variability. Based upon sequence similarities within sequences from core, E1 and NS5 regions, HCV is classified into 7 major genotypes (gt, 60%–70% sequence similarity) and numerous subtypes (75%–85% sequence similarity) [7].

During an acute infection with HCV only about 25% of the infected will clear the infection, while the majority will turn chronic [8]. One reason, why HCV is so successful in establishing a persistent infection, is evasion of and interference with the innate immune response that represents the first line of defence against, amongst others, viral infections [9].

HCV infects hepatocytes and is identified as non-self by intracellular pattern recognition receptors (PRRs) that activate the innate immune response. These PRRs bind to pathogen associated molecular patterns (PAMPs) that are accessible during the HCV replication cycle. The retinoic acid inducible gene-I (RIG-I) pathway is activated within hours after HCV infection, by binding of RIG-I to a RNA structure from the 3′ untranslated region of HCV and its replication intermediate [10,11]. The activated signaling cascade is composed of several steps including the involvement of the mitochondrial antiviral signaling protein (MAVS). In the end, the cascade leads to the activation of downstream effector molecules like the transcription factors nuclear factor κB (NFκB) and interferon regulatory factor (IRF)3 and switches the cell into an antiviral state [10].

Another PRR implicated in HCV recognition is Toll-like receptor (TLR)3, which is expressed in a number of liver-resident cell types, including hepatocytes and Kupffer cells (KCs) [12,13]. In contrast to RIG-I signaling, TLR3 signaling is induced a few days after HCV infection by the recognition of HCV dsRNA replication intermediates. The signal is transmitted by the TIR-domain-containing adaptor-inducing-interferon-β (TRIF) and activates IRF3 and NFκB [14,15].

Protein kinase R (PKR) is activated by binding to dsRNA at the internal ribosome entry site of HCV RNA. This leads to phosphorylation of the α subunit of eukaryotic initiation factor 2 (eIF2α) and the suppression of the translation of host mRNAs, while HCV translation continues from the HCV internal ribosome entry site. A kinase-independent signaling cascade via MAVS that drives the induction of interferon (IFN)-stimulated genes and IFN-β is also activated. The mechanisms of the crosstalk between PKR and RIG-I signaling are under investigation [16,17].

HCV interferes with the signaling pathways of the innate immune system at several steps. The viral protease NS3/4A is a central part of the evasion strategy as it cleaves not only the viral polyprotein but also MAVS, thereby preventing activation of the RIG-I pathway [18,19] and TRIF, the adaptor protein transmitting signals from TLR3 [20].

PKR seems to fulfill pro- and antiviral roles. While suppression of the translation of host mRNAs can inhibit the translation of type I IFN and IFN-inducible genes, it can also inhibit the translation of host factors necessary for HCV replication. Two HCV proteins, NS5A and E2, have been shown to inhibit the PKR kinase activity and thereby regulate the inhibition of the host mRNA translation [21–23]. The kinase-independent signaling pathway is like the RIG-I signaling pathway sensitive to the NS3/4A-mediated cleavage of MAVS.

The treatment of chronic HCV infection is based on pegylated IFN-α (pegIFN) and ribavirin (RBV) with different success rates. Achievement of a sustained virological response (SVR) is dependent on viral as well as host factors such as the viral genotype and the host IL-28B genotype [24]. For HCV gt 1 the first direct acting antivirals (DAAs), the NS3/4A protease inhibitors telaprevir and boceprevir, have been approved for treatment in combination with pegIFN and RBV in 2011. Triple combination therapy increased the SVR rate from around 40% up to 75% in treatment naive patients, but also the occurrence of adverse effects [25,26]. Recently, simeprevir, a second-generation NS3/4A protease inhibitor, has been approved for combination treatment of chronic HCV gt 1 infection. Together with pegIFN/RBV, simeprevir achieves SVR rates up to 90% in treatment naive patients, while the occurrence of adverse effects was not increased compared to pegIFN/RBV treatment alone [27]. End of 2013, sofosbuvir, the first nucleotide NS5B inhibitor, has been approved for treatment of chronic HCV infection. Sofosbuvir is also the first DAA approved for interferon-free treatment of patients chronically infected with HCV gt 2 and 3, which will significantly reduce adverse effects of HCV treatment [28]. Additional novel DAAs are currently in clinical trials and are expected to change the treatment of chronic HCV infection within the next years [29,30].

With time, 50% of those chronically infected with HCV will develop a chronic liver disease associated with e.g., fibrosis, and 5%–20% will progress to cirrhosis within 5–20 years. Every year, 1%–2% of these cirrhotic patients will develop HCC [4]. Whereas chronic hepatitis C can be treated, there is currently no treatment available for fibrosis other than organ transplantation. However, several antifibrotic compounds are under investigation that target the main signaling pathways involved in liver fibrosis such as the platelet derived growth factor and the transforming growth (TGF)-β signaling pathway [31]. One of these compounds is sorafenib, a receptor tyrosine kinase inhibitor that targets the platelet derived growth factor receptor and the Raf/Extracellular signal-regulated kinase signaling pathway. It has been approved for the treatment of HCC in 2007 and has also been shown to have antifibrotic effects [32–34]. For the treatment of HCC, there are currently no other drugs available; treatment alternatives are surgical removal of the tumor or organ transplantation.

Clearance of HCV infection during the acute phase has been associated with a strong and broad T cell response [35]. In chronic HCV infection, innate and adaptive immunity are impaired on many levels [36,37]. The HCV-mediated modulation of the chemokine response in the liver has implications for both viral persistence and the development of liver disease. In this review, we describe how HCV modulates the intrahepatic chemokine response, the relevance of these changes for liver disease progression and possibilities for targeting these changes for novel therapies.

### 1.2. Chemokines

Chemokines are a large family of small molecules (8–12 kD), the chemotactic cytokines. They are defined by a characteristic three-dimensional structure that is based on two disulphide bridges formed by four conserved cysteine residues. According to the relative position of the two *N*-terminal cysteine residues, chemokines are divided into four subfamilies: CXC, CC, CX3C and C. CXC and CC chemokines are the two major subfamilies, with the two *N*-terminal cysteines being separated by one amino acid or none, respectively. CX3CL1, also known as fractalkine or neurotactin, is the single member of the CX3C subfamily with 3 amino acids separating the first two cysteine residues. The C subfamily of chemokines is atypical in that it has only one *N*-terminal cysteine residue [38,39].

Previously chemokines have been divided according to their functionality into inflammatory and homeostatic chemokines. Inflammatory chemokines control the recruitment of leukocytes during infection, inflammation and tissue injury. They are strongly upregulated under inflammatory conditions and many have broad target-cell selectivity. Homeostatic chemokines are constitutively expressed and regulate homeostatic migration and homing of various cells. However several chemokines have been identified that fulfill both tasks; these have been termed “dual-function” chemokines. In contrast to the inflammatory chemokines, homeostatic and “dual-function” chemokines are highly selective [40].

While chemokines were discovered due to their chemotactic effect on leukocytes, they are now known to fulfill more roles than regulating (immune) cell migration and homeostasis. Chemokines are amongst others involved in the embryonic development of the central nervous system [41,42] and play a role in T cell development and activation [43,44]. Furthermore, they have angiogenic/angiostatic effects, which are of importance in cancer metastasis and present an option for the development of cancer therapies [45,46].

Another way chemokines differ from other cytokines is that their receptors belong to the superfamily of G-protein coupled receptors. As the chemokines, the receptors are grouped into four subfamilies, depending on the subfamily of their major chemokine ligand [47]. Upon activation, the chemokine receptor is phosphorylated and the heterotrimeric G protein dissociates from the receptor into its Gα- and Gβγ-subunit. The following signaling cascades induce conformational changes within the leukocyte integrins that enable the interaction of the cell with adhesion molecules on endothelial cells, such as intercellular adhesion molecule-1 and vascular cell adhesion molecule-1. After being secreted, chemokines can be immobilized by binding to glycosaminoglycans on the surface of endothelial cells and the extracellular matrix, thereby creating a gradient that coordinates trafficking of leukocytes to the target area [48].

Which leukocytes are recruited, depends on the chemokines secreted as well as on the chemokine receptors expressed on the surface of the target cell. Inflammatory chemokines often bind to more than one receptor and vice versa, but not necessarily have the same biological function. CXCL9, CXCL10 and CXCL11 e.g., are ligands of CXCR3 but antagonists of CCR3. The complexity of the chemokine network is further increased by the fact that one receptor can be expressed on various leukocyte subsets that might have divergent functions [49,50].

In addition to the conventional chemokine receptors a small family of atypical chemokine receptors (ACKR) has been identified that is comprised of at least four members; ACKR1 (Duffy antigen receptor for chemokines, DARC), ACKR2 (D6), ACKR3 (CXCR7) and ACKR4 (CCRL1). They are seven transmembrane-spanning receptors that resemble the conventional chemokine receptors structurally, but are unable to induce classical chemokine signaling pathways upon chemokine binding [51]. ACKR2 has been extensively characterised and has been shown to bind at least 12 inflammatory CC chemokines, including CCL2, CCL3, CCL4, CCL5, CCL7, CCL8 and CCL13. It has been implicated in the modulation of inflammatory responses by targeting these chemokines to cellular internalization and degradation [52,53]. In the liver this chemokine scavenging ability has been shown to reduce acute toxic liver injury [54].

In the liver, chemokines are not only involved in leukocyte trafficking but also act on and are secreted by liver resident cells like hepatocytes and hepatic stellate cells (HSCs), where they exert pro- as well as anti-fibrotic effects [55].

In the following section(s) we will focus on the chemokines that play a role during inflammatory responses in the liver.

## 2. Role of Chemokines in Intrahepatic Immunity

**Figure 1.**
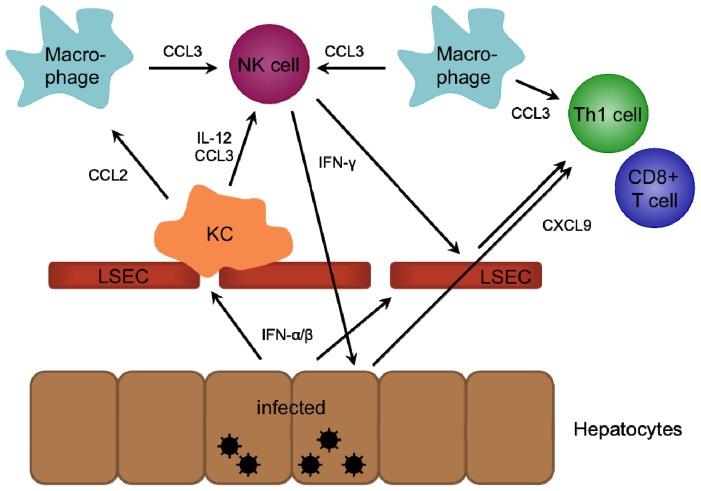
Recruitment of innate immune cells to the site of infection in the liver. Type I IFN released by hepatocytes upon pathogen recognition activates KCs, which secrete CCL2 and IL-12. CCL2 recruits macrophages, the main source of CCL3. NK cells recruited by CCL3 and activated by IL-12 release IFN-γ, that induces secretion of CXCL9 by LSECs and hepatocytes. CXCL9 and CCL3 attract Th1 and CD8+ T cells to the site of infection. DCs (not shown) are recruited by their CCR1/5 receptor and secrete amongst others CCL4 and CCL5. Neutrophils (not shown) are recruited by IL-1 or TNF-α-induced CXCL8 secretion from, e.g., KCs and enhance immune cell recruitment by secretion of CCL2, CXCL8, CXCL9 and CXCL10.

The liver is constantly exposed to food and microbial antigens from the gastrointestinal tract and therefore seems to have acquired specialized mechanisms that prevent an overactivation of the innate and adaptive immune system and induces a rather tolerogenic environment. This involves immune cells that can be only or to a higher extent found in the liver, including KCs, the liver-resident macrophages, natural killer (NK) and NKT cells. Furthermore, liver sinusoidal endothelial cells (LSECs), HSCs and hepatocytes can act as antigen presenting cells in addition to dendritic cells (DCs) and KCs [55–57]. This special environment also impacts the chemokine-induced recruitment of immune cells to the liver under inflammatory conditions. The chemokine receptors and chemokines, which are of importance in intra-hepatic immunity as well as their expression pattern and function are presented in Table 1 [38,48,58–113], whereas Figure 1 shows the mechanisms involved in the recruitment of innate immune cells to the infected liver.

### 2.1. CXCR3-Binding Chemokines

The CXCR3 chemokine receptor is associated with a type 1 helper T cell (Th1) response. It is predominantly expressed by Th1 cells, but also by CD8+ T cells, regulatory T cells (Tregs), NK cells, NKT cells, mast cells and HSCs [58,60,61]. The receptor is activated by the chemokines CXCL9, CXCL10 and CXCL11. In hepatocytes, recognition of PAMPs via the RIG-I or TLR3 signaling pathway activates transcription factors like NFκB and IRF3 that drive expression of CXCL10, amongst others. CXCR3-expressing CD8+ T cells, Th1 cells and NK cells that are recruited to the site of infection contribute to CXCL10 secretion by hepatocytes via the release of IFN-γ [96]. Expression of CXCL9 and 10 can be induced by IFN-γ and/or TNFα in several liver resident cells, including hepatocytes, LSECs, HSCs and KCs, promoting further recruitment of CXCR3-expressing cells to the site of infection [59,98,100]. CXCL11 is expressed by LSECs and is strongly induced by IFN-β and IFN-γ. It has a higher affinity for CXCR3 than CXCL9 and 10 and acts as a chemoattractant for activated T cells [97,99]. Additionally, neutrophils recruited to the site of infection produce numerous cytokines and chemokines, including CXCL9, CXCL10 and CXCL11 [101].

CXCL4 is together with CCL5 and CXCL7 one of the major chemokines stored in the α-granules of platelets. Release of chemokines by platelets is initiated upon activation by, e.g., thrombin, and is associated with the sealing of vascular wounds and tissue repair [46]. CXCR3-B, a splice variant of CXCR3, has been identified on human microvascular endothelial cells. It acts as a receptor for CXCL4, CXCL9, CXCL10 and CXCL11 and mediates the angiostatic effects of these chemokines [102]. CXCR3-B has also been detected on activated T cells but a chemotactic activity initiated by CXCL4 is disputed [46,103].

### 2.2. CXCR6-Binding Chemokines

The chemokine receptor CXCR6 is expressed by NKT cells, NK cells, activated CD8+ T cells and Th1 cells. CXCL16 is the only known ligand of CXCR6 and is expressed by LSECs and hepatocytes as well as cholangiocytes in the liver [113]. Additionally, CXCL16 is expressed by activated DCs and macrophages. Interaction with CXCL16 is important for the recruitment and retention of CXCR6 expressing effector cells to the liver. For NKT cells CXCR6 has not only been implicated in homing to the liver but also in antigen-dependent NKT cell activation via cell-cell contact with CXCL16-expressing activated DCs [111,112].

### 2.3. CCR1/5-Binding Chemokines

Whereas the chemokine receptor CCR1 is expressed on monocytes, immature DCs and NK cells, CCR5 expression is characteristic for Th1 cells but can also be found on monocytes, immature DCs, NK cells and other T cells subsets [58,60,61,64]. The chemokines CCL3 and 5 bind to both receptors. In contrast, CCL4 interacts only with CCR5 [38].

Infiltrating macrophages that are recruited by type I interferon-induced CCL2 secretion are the main source for CCL3 in the liver [63,67], but CCL3 is also secreted by activated KCs and plasmacytoid DCs (pDCs) [59,66]. Furthermore, KCs and endothelial liver cells also secrete CCL5 [62]. HSCs, another liver resident cell type, have been shown to secrete CCL3 and CCL4 upon TLR4-mediated activation [70]. While infiltrating myeloid DCs secrete more CCL3, pDCs secrete more CCL4 and CCL5 [66]. Additionally, non-classical CD14+ CD16+ monocytes that are recruited via CCR1 and CCR5 secrete inflammatory cytokines and chemokines including CCL3 and CCL4 [71].

CCR1 and CCR5 direct immature DCs to sites of inflammation via CCL3, CCL4 and CCL5. Maturation of DCs induced by, e.g., IL-1 or TNFα leads to the rapid loss of CCR1 and CCR5 surface expression and the upregulation of CCR7 on the cell surface. This enables the mature DC to migrate to lymphoid tissues in response to CCL21 for efficient antigen presentation [59,69].

CCL3 and CCL4 recruit NK cells to the site of infection, where they are activated by IL-12 and exert antiviral activity by secretion of IFN-γ [65,68]. Effector T cells are recruited via their CCR5 receptor in addition to chemotactic signals transmitted via their CXCR3 receptor. Depending on the strength of cell surface expression of these two chemokine receptors on different T cell subsets, they might be recruited to different intrahepatic compartments [114,115].

### 2.4. CCR4-Binding Chemokines

The chemokine receptor CCR4 is categorized as a dual-function chemokine receptor and its ligands CCL17 and CCL22 as dual-function chemokines, as they have roles both in inflammation and in homeostatic cell migration [38]. CCR4 is together with CCR8 a chemokine receptor characteristically expressed by type 2 helper T (Th2) cells. Furthermore, CCR4 is expressed by skin homing and regulatory T cells [80,82,83].

CCL17 and CCL22 can be secreted by DCs, macrophages and monocytes. CCL22 is constitutively secreted by DCs and macrophages, while CCL22-secretion by monocytes is induced by stimulation with IL-4 or IL-13 and inhibited by IL-10. DCs stimulated with TLR ligands like LPS, CpG or poly(I:C) strongly upregulate CCL17 expression. Additionally, secretion of CCL17 by DCs or monocytes is also stimulated by IL-4. Thus a Th2 cytokine milieu stimulates the secretion of CCL17 and CCL22 leading to further recruitment of Th2 cells and Tregs [78,79,81].

### 2.5. CCR2-Binding Chemokines

The only known receptor for the chemokine CCL2 is the chemokine receptor CCR2 that is predominantly expressed by monocytes, but also by Th1 and Th2 cells and pDCs [48,58]. CCL2 is involved in the recruitment of monocytes, memory T cells and NK cells *in vitro* and it predominantly recruits monocytes *in vivo* [72,74,76,77]. Furthermore, CCL2 has been shown to be necessary for the differentiation of CD4+ T cells into Th2 cells [75].

In the liver, the release of IFN-α/β in response to pathogen recognition induces the secretion of CCL2 by KCs, which leads to the CCR2-dependent recruitment of monocytes and macrophages within 48 h. These infiltrating macrophages are the main source for CCL3 that is involved in the recruitment of NK cells [63,67]. CCR2 expressing pDCs that infiltrate the liver in response to CCL2 also secrete a number of chemokines that are involved in the recruitment of effector T cells (CXCL9, CXCL10, CXCL11) as well as monocytes and immature DCs (CCL3, CCL4, CCL5) [66,73]. Additionally, LPS signaling via TLR4 sensitizes HSCs to TGF-β-induced activation by KCs. Activated HSCs secrete various chemokines, including CCL2 and CCL3, and thereby further enhance the infiltration of monocytes [61,70].

### 2.6. CXCR1/2-Binding Chemokines

The chemokine receptors CXCR1 and CXCR2 are predominantly expressed by neutrophils but can also be found on the surface of monocytes and mast cells [58,60]. CXCR1 has recently been shown to be upregulated on hepatocytes in response to liver injury and CXCR1 positive CD8+ T cells responding to CXCL8 have been detected in different viral infections [85–87].

Both receptors bind CXCL8 with high affinity but have a different selectivity for other CXC chemokines [84]. CXCL8 is a strong chemoattractant for neutrophils and can be induced by IL-1 or TNFα in a variety of cells [88,89,92]. In the liver, CXCL8 expression has been shown in LSECs, KCs and hepatocytes upon stimulation. Additionally, CXCL8 can be produced by Th1 and Th17 cells [90,91,93]. Expression of CXCL8 is not only induced by IL-1 or TNFα but also by viral infection or poly(I:C). This direct induction of CXCL8 is dependent on RIG-I signaling and the downstream activation of IRF3 and NFκB, which bind directly to the CXCL8 promotor [94,95].

Neutrophils that are primarily recruited by CXCL8 can exert antiviral immunity amongst others by the release of inflammatory cytokines (e.g., IL-12, TNFα) and chemokines (e.g., CCL2, CXCL8, CXCL9, CXCL10) that leads to further recruitment of immune cells to the site of infection [101].

### 2.7. CXCR4/7-Binding Chemokines

CXCR4 is classified as homeostatic chemokine receptor and is as such abundantly expressed in leukocytes and most human tissues. CXCR4 binds CXCL12 and the non-chemokine ligands ubiquitin and macrophage migration inhibitory factor (MIF). As a homeostatic receptor-chemokine-pair, CXCR4-CXCL12 plays amongst others an important role in hematopoiesis, development and organization of the immune system [104–106,110]. In the liver, CXCR4 expression by HSCs has been detected and is upregulated upon HSC activation. Furthermore, it has been shown that CXCL12 activates HSCs that then secrete CXCL12, which leads to further activation and proliferation, which has implications for the development of fibrosis [107].

CXCL12 is also a ligand for CXCR7 (ACKR3) a member of the family of atypical chemokine receptors. The affinity of CXCL12 for CXCR7 is higher than for CXCL4 and CXCR7 can modulate the effects of CXCL12 by heterodimerization with CXCR4. Due to their role in neoangiogenesis, the expression and interaction of CXCR4, CXCR7 and CXCL12 has been associated with a variety of tumors [105,108,109].

## 3. HCV-Mediated Modulation of the Chemokine Response

To be able to fight the virus, antiviral immune cells need to be recruited to the site of the infection. Chemokines play an important role in the chemotaxis of these immune cells. Thus, interference with the expression of chemokines or chemokine receptors would allow HCV to evade host immunity and to establish viral persistence.

Once HCV infection is established, recruited immune cells are responsible for chronic inflammation and liver damage, which may result in further liver disease such as liver fibrosis, cirrhosis and cancer. The attenuation of intrahepatic immunity during the chronic phase of the infection would thus increase the survival of both the host and the virus.

**Figure 2.**
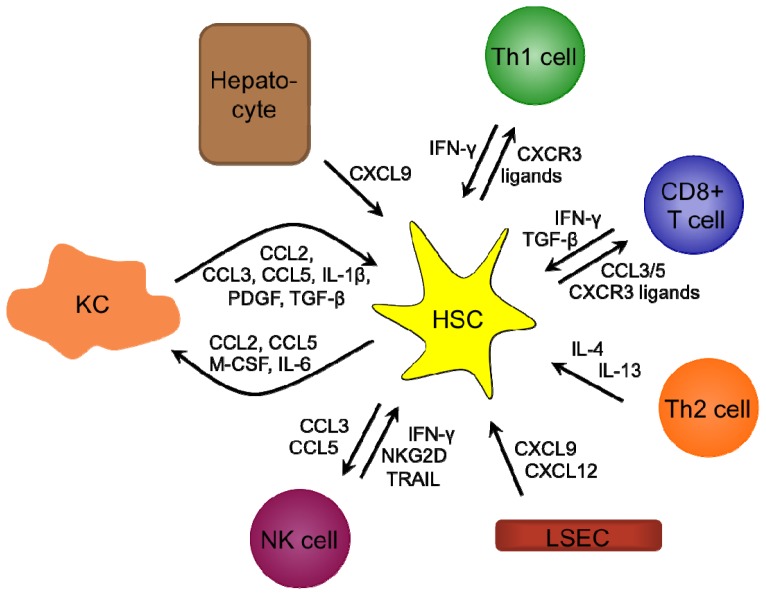
Modulation of hepatic stellate cells (HSCs) during chronic hepatitis C. HSCs are involved in chronic hepatitis C by recruiting immune cells through the secretion of chemokines such as CCL2, CCL5, CXCL9 and CXCL10. KCs contribute to HSC activation by producing CCL2, CCL3, CCL5 and TGF-β, T cells by producing TGF-β and LSECs by secreting CXCL12. CXCL9 produced by hepatocytes and LSECs and IFN-γ produced by NK and T cells are antifibrogenic. In addition, NK cells are antifibrogenic by expressing NKG2D and TRAIL. Activated HSCs proliferate (induced, e.g., by PDGF) and produce collagen (induced e.g., by IL-4, IL-13 and TGF-β) resulting in liver fibrosis.

The HCV-mediated modulation of the chemokine response is discussed in the following section. Figure 2 shows how HSCs, which are the key effector cells of liver fibrogenesis, are modulated during chronic hepatitis C, whereas Table 2 summarizes the HCV-mediated changes in the expression of chemokines and chemokine receptors mentioned in this review [116–136].

### 3.1. CXCR3-Dependent Lymphocyte Recruitment in HCV Infection

Since Th1 cells, cytotoxic CD8+ T cells and NK cells, which play a major role in the defense against viruses, express the chemokine receptor CXCR3, the modulation of CXCR3 expression or of its ligands would represent an efficient viral escape mechanism.

During the initial stages of HCV infection, the expression of CXCR3 ligands such as CXCL10 in infected hepatocytes is caused by TLR3 and RIG-I activation and is further amplified by type I and III IFN induction [137]. However, when HCV replication is established, several HCV proteins interfere with PRR activation and IFN signaling [36]. The HCV NS3/4A protease in particular has been shown to block RIG-I and TLR3 signaling through cleavage of MAVS and TRIF [18–20]. Thus, during acute HCV infection CXCR3-associated chemokines start to increase only several weeks after virus acquisition, suggesting a role for cellular immune responses in chemokine induction [138].

In chronic hepatitis C patients, the levels of the three CXCR3 ligands CXCL9, CXCL10 and CXCL11 are increased in the liver [132,133,139]. Similarly, experimentally infected chimpanzees are characterized by enhanced intrahepatic levels of CXCL10 and CXCL11 [133]. This is paralleled by a high frequency of CXCR3 positive CD8+ T cells during chronic HCV infection [132,134]. In addition, a CXCL11 promoter polymorphism resulting in decreased CXCL11 expression is more frequent in chronic HCV patients than in healthy controls, emphasizing the importance of CXCR3 positive lymphocytes for HCV clearance [140]. However, although CXCR3 positive T cells may promote HCV clearance in early phases of the infection, they cause tissue injury when the infection persists. It is likely that the CXCR3 positive T cells recruited in the chronically infected liver include both HCV-specific and bystander T cells, since both are thought to contribute to liver injury [141]. Thus, HCV may benefit from limiting the recruitment of CXCR3 positive immune cells by extending the survival of the host. This may explain why liver-specific NS3/4A expression decreases the intrahepatic levels of CXCL9, which diminishes the recruitment of Th1 cells [120,125]. Additionally, NS3/4A blocks the virus-induced transcriptional activation of the CXCL10 promoter [135]. Thus, without the HCV-mediated modulation of CXCR3 ligand expression, liver damage induced by non-specific CXCR3 expressing immune cells may be even higher.

### 3.2. Involvement of CXCR6 Positive Immune Cells in HCV Infection

In the livers of chronic hepatitis C patients, CXCR6 is expressed on CD4+ T cells, CD8+ T cells, NK, NKT and B cells, while the CXCR6 ligand CXCL16 is produced by hepatocytes and bile ducts [113,142]. Pretreatment CXCL16 level increase significantly during IFN therapy, suggesting that IFN treatment induces the recruitment of CXCR6 positive immune cells [136]. Most effector T cells infiltrating the chronically inflamed human liver express not only high levels of CCR1, CCR5 and CXCR3 but also CXCR6, so that CXCL16 expression is important for efficient T cell homing to the liver. Recently, a unique subset of HCV-specific CXCR6+ liver-infiltrating CD8+ T cells that express the C-type lectin CD161 and secrete IL-17 and IFN-γ has been described [143]. Furthermore, CXCR6 is also required for the hepatic homing of NK and NKT cells [111].

### 3.3. Recruitment of CCR1/5 Positive Lymphocytes in HCV Infection

CCR1 and CCR5 are expressed by Th1 cells, CD8+ T cells, memory T cells and NK cells. Since CCR5 positive T cells are an integral part of antiviral immunity, a high frequency of these cells can be detected during acute HCV infection [127]. During chronic hepatitis C, the intrahepatic expression of the CCR5 ligands CCL3, 4 and 5 is increased [116,139,144,145]. Whereas HCV core and NS5A have been shown to increase CCL5 expression in cell culture, NS3/4A exerts an inhibitory effect on CCL5 expression [118,119], suggesting that the different HCV proteins have opposing effects on the expression of CCL5. *In vivo*, liver-specific expression of NS3/4A resulted in a decrease of intrahepatic CCL3 levels [120], supporting the anti-inflammatory role of NS3/4A.

Since the expression of chemokines is cell-specific, the pattern of chemokine secretion in the infected liver of chronic hepatitis C patients is guiding the distribution of T cells to different intrahepatic compartments. While CCR5 ligands are predominantly expressed by vascular endothelium within portal tracts, CXCR3 ligands are amongst others expressed by hepatocytes and sinusoidal endothelium [114,139,145,146]. Interestingly, the proportion of CD8+ T cells with CCR1 and CCR5 surface expression is decreased in chronic hepatitis C patients, suggesting that chronic HCV infection induces receptor internalization [117]. This mechanism may contribute to HCV persistence and pathogenesis. The importance of the CCR5 axis for HCV clearance was shown in studies analyzing CCR5 gene polymorphisms. Patients bearing the mutation CCR5-Δ32 that abrogates CCR5 expression have a higher HCV prevalence and are characterized by increased viral loads when infected with HCV [147].

### 3.4. Hepatic Homing of CCR4 Positive Lymphocytes in HCV Infection

T cell subpopulations such as Th2 cells and Tregs express the chemokine receptor CCR4. Since these cell types interfere with antiviral immunity, the modulation of CCR4 ligands may contribute to viral persistence. Indeed, the expression of the CCR4 ligands CCL17 and CCL22 is enhanced in chronic hepatitis C patients [125,126]. It has been shown that contact of DCs with HCV-infected cells strongly stimulates the expression of CCL17 and CCL22, which act as attractants for Tregs [126]. The HCV protein mediating this effect may be NS3/4A, since mice with liver-specific expression of NS3/4A are characterized by enhanced intrahepatic levels of CCL17 and CCL22 resulting in increased numbers of CCR4 positive CD4+ T cells in the livers of NS3/4A-transgenic mice [120,125]. The HCV-mediated cleavage of the T cell protein tyrosine phosphatase is hereby of major importance [120,148,149].

### 3.5. CCR2-Dependent Recruitment of Monocytes and Macrophages in HCV Infection

A central factor responsible for macrophage activation and recruitment of monocytes and macrophages to the liver is the chemokine CCL2. In the early phase of the infection, cellular antiviral immunity is initiated by Kupffer cells releasing CCL2, resulting in the recruitment of CCL3-producing monocytes and pDCs, which attract NK cells. Thus, the expression of CCL2 and CCR2 is increased in HCV-infected patients [121,122]. Interestingly, intrahepatic CCL2 expression was further increased in obese HCV-infected subjects as compared to lean HCV-infected subjects, possibly contributing to the faster progression of liver disease in obese subjects with chronic hepatitis C [145]. We found that liver-specific expression of NS3/4A increases the intrahepatic levels of CCL2 and TNF-α, which is paralleled by an enhanced intrahepatic number of macrophages [123]. In addition, NS5A could augment the expression of CCL2 in cell culture [124]. Thus, NS3/4A and NS5A may induce a CCL2-mediated recruitment of macrophages to the liver resulting in increased TNF-α levels.

### 3.6. CXCR1/2-Expressing Immune Cells during HCV Infection

The CXCR1/2 ligand CXCL8 is involved in the recruitment of neutrophils and monocytes to the liver. Since these cell types are involved in antiviral immunity, the levels of CXCL8 are increased in HCV-infected patients compared to healthy controls [128,129]. When the infection persists, CXCL8 expression correlates with the severity of inflammation [150]. Interestingly, elevated levels of CXCL8 in the serum are also associated with resistance to interferon therapy [129]. Several HCV proteins seem to modulate CXCL8 expression. While HCV core, NS4A, NS4B and NS5B induce the expression of CXCL8, NS3/4A decreases CXCL8 expression [119,130,131].

### 3.7. Liver Fibrosis

When untreated, HCV-induced chronic hepatitis can progress to hepatic fibrosis and ultimately cirrhosis and HCC. Liver fibrosis/cirrhosis is characterized by an excessive accumulation of extracellular matrix leading to the formation of hepatic scar tissue. Activated HSCs and portal fibroblasts are the key effector cells of liver fibrogenesis [151]. Chemokines are involved in liver fibrosis induction both by recruiting inflammatory immune cells and by affecting HSCs. Multiple immune cell types such as monocytes/macrophages, neutrophils, NK cells, NKT cells, B cells and T cells are involved in the development of liver fibrosis [151].

CCL2 is the main chemokine responsible for the intrahepatic recruitment of monocytes and macrophages and crucial for the initiation of liver fibrogenesis [152]. In patients infected with HCV, CCL2 levels are increased [122] and the expression of CCR2 is correlated with the fibrosis stage [121]. Thus, a CCL2 promoter polymorphism at position −2578, which is associated with increased CCL2 production, may predispose HCV patients to more severe hepatic inflammation and fibrosis [153].

Furthermore, the receptor CCR5 is involved in fibrogenesis. It has been shown that human HSCs express CCR5 and that CCR5 activation stimulates HSC migration and proliferation [154]. In addition, neutralization studies identified important roles for both CCR1- and CCR5-expressing macrophages in the development of liver fibrosis [155]. Importantly, the intrahepatic levels of the CCR5 ligands CCL3, 4 and 5 are increased in chronic hepatitis C [116].

While neutrophils, NKT cells and B cells are mainly profibrogenic, NK cells seem to have an antifibrotic role. NK cells ameliorate liver fibrosis by killing activated HSCs in a NKG2D- and TRAIL-dependent manner [156]. Since the frequency of NK cells is reduced in the liver of chronic hepatitis C patients [157], the augmentation of chemokine mediated NK cell trafficking to the liver may be an option to treat liver fibrosis.

HCV-derived CD8+ T cells were shown to contribute to HSC activation by an increase in TGF-β and a decrease in IFN-γ expression [158]. Th1 cells are involved in chronic hepatitis by producing proinflammatory cytokines such as IFN-γ. After the initiation of liver fibrosis, IFN-γ is antifibrotic by inducing HSC apoptosis and cell cycle arrest [159]. In this setting, Th2 cells are profibrotic by stimulating collagen synthesis [160]. Interestingly, liver-specific expression of NS3/4A induces an increase in intrahepatic CCL17 and CCL22, which is paralleled by a decrease in CCL3, CXCL9 and CXCL11 resulting in a switch of the Th1/Th2 ratio towards CCR4+ Th2 cells [120,125].

### 3.8. Liver Cancer

HCV-infected patients with chronic hepatitis and liver fibrosis/cirrhosis are at a high risk of developing HCC. In these patients, repeated cycles of immune-mediated destruction of infected hepatocytes and virus-induced apoptosis together with regeneration of damaged tissue cause disturbances in the normal cellular homeostasis of the liver. While the chemokine-driven infiltration of lymphocytes contributes to cancer development, the recruitment of tumor-specific immune cells is beneficial once the tumor has established. In this case, the expression of CCL5 and CXCL10, which drive tumor infiltration by T and NK cells correlates with patient survival [161]. Similarly, poor survival is associated with an increased intrahepatic frequency of immunosuppressive cell types such as myeloid-derived suppressor cells (MDSCs) and Tregs [162,163].

## 4. Chemokine Signaling as Therapeutic Target for Liver-Related Diseases

Since chemokines play an important role in the development of HCV-induced liver disease, inhibition or enhancement of chemokine signaling constitutes an attractive therapy option.

### 4.1. Acute Liver Injury

Several chemokine receptors such as CCR1, CCR2, CCR5, CXCR1, CXCR2, CXCR3, CXCR6 and D6 (ACKR2) are involved in the pathophysiology of acute liver injury mainly by regulating the recruitment of specific immune cell populations.

The chemokine receptor CCR1, which is activated by ligands such as CCL3 and CCL5, has been shown to be involved in the enhancement of acute liver disease. Mice deficient for CCL3 are resistant towards concanavalin A-induced acute liver failure, since the recruitment of CCR1-positive CD4+ T cells is decreased in these mice [164]. In the same model, activation of CCR1 by CCL5 resulted in the intrahepatic infiltration of NK cells causing hepatocyte damage [165]. In contrast, deficiency of CCR5, which shares the ligands CCL3 and CCL5 with CCR1, exerbated T cell-mediated hepatitis in mice. This effect may be mediated by compensation of CCR5-induced effects by CCR1 [166].

The role of CCR2 in acute liver injury can be either harmful or beneficial depending on the used mouse model. Mice deficient for CCR2 are characterized by reduced liver damage and macrophage accumulation after acute carbon tetrachloride injection [167]. Furthermore, treatment with the CCR2 inhibitor propagermanium was able to improve liver injury induced by concanavalin A, Corynebacterium parvum and lipopolysaccharide (LPS) injection [168,169]. However, acetaminophen-induced liver damage was increased in CCR2-deficient mice, most probably by preventing macrophage-mediated neutrophil apoptosis and phagocytosis of cell debris [170].

In addition, it was shown that the chemokine scavenger receptor D6 is limiting acute toxic liver injury. Mice deficient for D6 were characterized by increased infiltration of NK and T cells resulting in enhanced liver damage after acute carbon tetrachloride treatment [54]. Furthermore, genetic variations of D6 contribute to the grade of liver inflammation in patients infected with HCV [171].

The receptors CXCR1 and CXCR2 are involved in neutrophil-induced acute inflammatory liver injury. High levels of the CXCR1/CXCR2 ligand CXCL8 in the liver and the circulation have been found in patients with acute liver injuries such as alcoholic hepatitis or ischemia-reperfusion injury [172]. Furthermore, CXCR2 deficient mice had significantly less liver injury after ischemia/reperfusion than control mice [173]. Importantly, treatment with the CXCR2 antagonist SB225002 [173] or the CXCR1/2 inhibitor repertaxin [174] was able to prevent reperfusion injury.

Interestingly, mice deficient for CXCR3 developed a more severe liver injury with higher plasma transaminase levels and a more pronounced Th1/Th17 response compared with control mice when treated with concanavalin A. This effect was caused by decreased recruitment of IL10-producing CXCR3+ Tregs [175]. These results are in line with a study analyzing the role of CXCR3 in carbon tetrachloride-induced acute liver injury [176]. However, since studies investigating ischemia-reperfusion injury [177] or adenovirus-induced liver injury [178] describe harmful effects mediated by CXCR3, the role of CXCR3 in acute liver injury seems to be dependent on the applied mouse model.

Recently, it was shown that NKT cells exert proinflammatory effects and promote acute liver injury in a CXCR6-dependent way [179]. Furthermore, inhibition of the CXCR6 ligand CXCL16 by neutralizing antibodies was able to decrease liver injury induced by Bacille Calmette-Guerin and lipopolysaccharide injection [180], indicating the important role of the CXCL16-CXCR6 axis in the induction of acute liver injury.

Thus, blockade of CCR1, CXCR2 and CXCR6 as well as activation of D6 may be beneficial in the treatment of acute fulminant hepatitis.

### 4.2. Metabolic Syndrome

The CCL2-CCR2 axis also seems to be involved in the modulation of liver steatosis and diabetes. CCL2 was enhanced both in the liver and the serum of diabetic mice, which was paralleled by hepatic accumulation of macrophages. Interestingly, treatment with the CCR2 antagonist RS504393 was able to ameliorate hyperglycaemia, liver steatosis and liver inflammation [181]. Similarly, treatment with the CCR2 inhibitor propagermanium could improve diet-induced metabolic disorders such as insulin resistance and liver steatosis [182]. Thus, interruption of the CCL2/CCR2 pathway may provide an effective therapeutic strategy for attenuating the progression of liver steatosis and of associated metabolic disorders.

### 4.3. Liver Fibrosis

By using different mouse models for liver fibrosis such as bile duct ligation or treatment with carbon tetrachloride, it was shown that both CCR1 and CCR5 promote hepatic fibrosis [155]. By contrast, liver fibrosis development in MDR2 deficient mice is only dependent on CCR5 but not on CCR1 [183], thus confirming the role of CCR5 as promotor of liver fibrosis. However, the relevance of CCR1 for fibrosis induction remains unclear.

In addition, the CCR2 ligand CCL2 is involved in liver fibrosis development by recruiting CCR2-expressing inflammatory macrophages [184,185]. However, while treatment with the CCL2 inhibitor mNOX-E36 resulted in a decrease of monocyte/macrophage infiltration and hepatic steatosis in the murine models of carbon tetrachloride- and MCD diet-induced hepatic injury, mNOX-E36 application was not able to alter fibrosis progression [186].

Since profibrogenic functions of HSCs are induced through CXCR2 [187] and the CXCR2 ligand CXCL8 is associated with hepatic macrophage accumulation in human liver fibrosis [188], CXCR2 evolved as an attractive target for liver fibrosis treatment.

The CXCR3 ligands CXCL4, CXCL9 and CXCL10 seem to have divergent functions in liver fibrosis. While CXCL4 and CXCL10 are profibrogenic, CXCL9 has been shown to exert mainly antifibrotic effects. CXCL4-deficient mice treated with carbon tetrachloride or thioacetamide developed a less severe liver fibrosis as compared to control mice, which may be explained by a reduced hepatic infiltration of CD8+ T cells [189]. Similarly, CXCL10-deficient mice showed massively reduced liver fibrosis upon carbon tetrachloride administration by inducing the inactivation of HSCs by NK cells [190]. In contrast, CXCL9 represses collagen expression in HSCs and is antifibrotic in humans and mice [191].

Furthermore, it was shown that HSCs express the chemokine receptor CXCR4 and that CXCR4 activation by CXCL12 is profibrogenic by increasing HSC activation and proliferation [107]. *In vivo*, fibrosis development is regulated by the balance between the CXCL12 receptors CXCR4 and CXCR7. While after acute injury pro-regenerative CXCR7 signaling is activated, chronic liver injury results in loss of CXCR7 and upregulation of profibrotic CXCR4 [192]. Interestingly, a CXCR4 antagonist (plerixafor) is already approved for the treatment of non-Hodgkin lymphoma and multiple myeloma.

In addition, CXCR6 promotes hepatic fibrogenesis, since CXCR6-deficient mice were protected from liver fibrosis progression in two different mouse models. CXCR6-expressing NKT cells were found to induce hepatic inflammation and to recruit macrophages resulting in liver fibrosis [179].

Thus, CCR5, CXCR2, CXCR4 and CXCR6 antagonists or the application of CXCL9 may serve for prevention and treatment of liver fibrosis.

### 4.4. Liver Cancer

The receptors CCR1 and CCR5 play also crucial roles in tumorigenesis. Whereas HCC induced by N-nitrosodiethylamine treatment occurred more frequently in both CCL3- and CCR1-deficient mice compared with control mice, tumor burden was dramatically reduced by CCL3 or CCR1 deficiency [193]. The critical role of CCR1 in tumor progression was confirmed in the MDR2 knockout model, in which mice deficient for CCR1 and MDR2 had smaller tumors than MDR2 knockout mice [183]. In contrast to CCR1, CCR5 promotes both the development and progression of liver cancer [183], suggesting that CCR5 antagonists may be very useful in HCC prevention and therapy.

Selective recruitment of immune cells with anti-inflammatory functions such as MDSCs and Tregs is one of the mechanisms of tumor-driven immune evasion as they suppress T cell and NK cell mediated anti-tumor functions. An increase in MDSC frequency has been shown both in patients with HCC [163] and in different HCC mouse models [194]. Thus, targeting of MDSC, which express CXCR2 might be an option to treat HCC. Interestingly, treatment with α-CXCL1 was able to reduce the intrahepatic MDSC frequency in mice with HCC [194].

An increase in Treg frequency was found in both peripheral blood and tumor margin of HCC patients, which correlates with poor survival [162]. Thus, blocking of pathways involved in Treg recruitment would be an option to decrease the frequency of Tregs in the tumor tissue and prevent immune escape in HCC. Major chemokine receptors involved in the migration of Tregs to the liver are CCR4, CCR6 and CXCR3. Unfortunately, these chemokine receptors are also expressed by immune cells involved in anti-tumor immunity. CXCR3 e.g., is also highly expressed on NK, Th1 and Th17 cells.

Recently, it was shown that the CXCL12/CXCR4 pathway is involved in tumor-associated fibrosis by increasing myofibroblast infiltration and differentiation [195]. Addition of the CXCR4 antagonist AMD3100 to sorafenib in an HCC mouse model prevented the increase in tumor-associated fibrosis and significantly inhibited HCC growth compared to sorafenib alone [195]. Besides of CXCR4, CXCL12 can bind to CXCR7. CXCR7 is overexpressed in liver cancer tissue and positively regulates the invasion, angiogenesis and tumor growth of human hepatocellular carcinoma cells [196].

## 5. Conclusions

The successful introduction of the CCR5 antagonist maraviroc in HIV therapy [197] suggests that the modulation of chemokine signaling may be feasible for the therapy of HCV-mediated diseases as well.

However, it should always be taken into account that the blockade of antiviral immune cells may result in a rebound of virus titers. This may be solved by combining immunotherapies (directed, e.g., against liver fibrosis) with direct antivirals. The stage of the disease also plays a role. While a dampening of the intrahepatic immune response may be beneficial during acute or chronic hepatitis, a strengthening of the anti-tumoral immune response during HCC is the aim.

One further concern is the redundancy of the chemokine system. Most of the receptors can bind more than one ligand and several chemokines can signal through more than one receptor. Thus, the blockade of one receptor or ligand may cause compensatory effects such as the upregulation of other receptors or ligands resulting in undesired side effects.

In addition, there are differences in the expression of chemokines and chemokine receptors between mice and humans, complicating the translation of mouse data to the clinic. While the receptor CCR1 is constitutively expressed by neutrophils in mice [198], CCR1 is a major receptor for monocyte recruitment in humans and has only a low expression in neutrophils [199].

Furthermore, most chemokine receptors are expressed by different immune cells. One example is CXCR3, which is expressed by Th1 cells, CD8+ T cells, Tregs, NK cells, NKT cells and HSCs. While the treatment of CXCR3-deficient mice with concanavalin A results in a decreased recruitment of IL10-producing CXCR3+ Tregs [175], CXCR3 blockade in a model for ischemia-reperfusion injury causes a reduction in intrahepatic Th1 cells [177].

Thus, targeting of the chemokine system requires a careful selection of the appropriate target. The stage of the disease and possible discrepancies between the murine and the human system have to be taken into account. However, the approval of the CCR5 antagonist maraviroc and the CXCR4 antagonist plerixafor as well as ongoing clinical trials against diverse diseases such as rheumatoid arthritis, chronic obstructive pulmonary disease or Crohn’s disease show the great potential of therapies modulating the chemokine response.

## Figures and Tables

**Table 1.** Summary of the chemokine receptors and their ligands important in intra-hepatic immunity.

Chemokine receptor	Expression on	Ligands	Secretion by	Function	References
CCR1	immature DCs, monocytes, memory T cells, NK cells	CCL3, CCL5	infiltrating macrophages, KCs, DCs, activated HSCs, LSECs	recruitment of NK cells, immature DCs	[38,58–71]
CCR2	monocytes, memory T cells, pDCs	CCL2, CCL7, CCL8, CCL13	KCs, activated HSCs	recruitment of monocytes	[48,58,61,63,66,67,70, 72–77]
CCR4	Th2 cells, Tregs	CCL17, CCL22	DCs, macrophages, monocytes	recruitment of Th2 cells, Tregs	[38,78–83]
CCR5	immature DCs, Th1 cells, NK cells, monocytes, pDCs, HSCs	CCL3, CCL4, CCL5,	infiltrating macrophages, KCs, DCs, activated HSCs, LSECs	recruitment of NK cells, immature DCs, effector T cells	[38,58–71]
CXCR1	neutrophils, monocytes, mast cells, hepatocytes	CXCL6, CXCL7, CXCL8,	LSECs, KCs, activated hepatocytes, Th1 cells, Th17 cells	recruitment of neutrophils	[58,60,84–95]
CXCR2	neutrophils, monocytes, mast cells	CXCL1-3, CXCL5, CXCL6, CXCL7, CXCL8	LSECs, KCs, activated hepatocytes, Th1 cells, Th17 cells	recruitment of neutrophils	[58,60,84,87–95]
CXCR3	CD8 T cells, Th1 cells, NK cells, NKT cells, Tregs, HSCs, mast cells	CXCL9, CXCL10, CXCL11,	hepatocytes, LSECs, HSCs, KCs, DCs, neutrophils	recruitment of NK, NKT and T cells	[58–61,96–101]
CXCR3-B	microvascular endothelial cells, activated T cells	CXCL4, CXCL9/10/11	platelets	mediation of angiostatic effects	[46,102,103]
CXCR4	widely expressed, HSCs	CXCL12	activated HSCs	neoangiogenesisand HSC activation	[104–110]
CXCR6	Th1 T cells, CD8 T cells, NKT cells, NK cells	CXCL16	LSECs, hepatocytes, cholangiocytes, activated DCs	recruitment and retention of effector cells in the liver	[111–113]

**Table 2.** Summary of the HCV-mediated changes in the expression of chemokines and chemokine receptors mentioned in this review.

Chemokine receptor	Ligands	Changes in HCV infection	References
CCR1	CCL3, CCL5, CCL7, CCL8, CCL13-16, CCL23	Increased intrahepatic CCL3 and 5 expression during chronic hepatitis C	[116]
		Decreased proportion of CCR1+ CD8+ T cells in chronic hepatitis C patients	[117]
		Increase of CCL5 expression by HCV core and NS5A *in vitro*, but decrease by NS3/4A	[118,119]
		Decrease of the intrahepatic level of CCL3 by NS3/4A *in vivo*	[120]
CCR2	CCL2, CCL7, CCL8, CCL13	Increased expression of CCL2 and CCR2 in HCV-infected patients	[121,122]
		Increase of CCL2 expression by HCV NS3/4A and NS5A	[123,124]
CCR4	CCL17, CCL22	Enhanced expression of CCL17 and CCL22 in chronic hepatitis C patients	[125,126]
		Increased intrahepatic levels of CCL17 and CCL22 through liver-specific HCV NS3/4A expression	[125]
CCR5	CCL3-5, CCL8	High frequency of CCR5+ T cells during acute HCV infection	[127]
		Increased CCL3, 4 and 5 expression during chronic hepatitis C	[116]
		Decreased proportion of CCR5+ CD8+ T cells in chronic hepatitis C patients	[117]
		Increase of CCL5 expression by HCV core and NS5A *in vitro*, but decrease by NS3/4A	[118,119]
		Decrease of the intrahepatic level of CCL3 by NS3/4A *in vivo*	[120]
CXCR1/2	CXCL6-8 (for CXCR1) and CXCL1-8 (for CXCR2)	Increased levels of CXCL8 in HCV-infected patients	[128,129]
		Induction of CXCL8 expression by HCV core, NS4A, NS4B and NS5B, but decrease by NS3/4A	[119,130,131]
CXCR3	CXCL4, CXCL9-11	Increased intrahepatic CXCL9, CXCL10 and CXCL11 levels during chronic hepatitis C	[132]
		Increased intrahepatic CXCL10 and CXCL11 levels in HCV-infected chimpanzees	[133]
		High frequency of CXCR3+ CD8+ T cells during chronic HCV infection	[132,134]
		Decrease of the intrahepatic levels of CXCL9 and 11 by NS3/4A *in vivo*	[125]
		Reduction of CXCL10 expression by NS3/4A *in vitro*	[135]
CXCR6	CXCL16	Increase in CXCL16 levels during IFN therapy	[136]

## Abbreviations

ACKR: atypical chemokine receptor
DAA: direct acting antiviral
DC: dendritic cell
gt: genotype
HCC: hepatocellular carcinoma
HCV: hepatitis C virus
HSC: hepatic stellate cell
IFN: interferon
IL: interleukin
IRF: interferon regulatory factor
LPS: lipopolysaccaride
LSEC: liver sinusoidal endothelial cell
MAVS: mitochondrial antiviral signaling protein
MDSC: myeloid-derived suppressor cell
NFκB: nuclear factor κB
KC: Kupffer cell
NK cell: natural killer cell
NS: non-structural
pegIFN: pegylated interferon-α
PAMP: pathogen associated molecular pattern
pDC: plasmacytoid dendritic cell
PKR: protein kinase R
PRR: pattern recognition receptor
RBV: ribavirin
RIG-I: retinoic acid inducible gene-I
TCPTP: T cell protein tyrosine phosphatase
TGF: transforming growth factor
Th: T-helper
TLR: Toll-like receptor
TNF: tumor necrosis factor
TRIF: TIR-domain-containing adaptor-inducing-interferon-β
Treg: regulatory T cell
